# Effectiveness of a Community Health Worker-Led Diabetes Intervention among Older and Younger Latino Participants: Results from a Randomized Controlled Trial

**DOI:** 10.3390/geriatrics3030047

**Published:** 2018-08-02

**Authors:** Barbara Mendez Campos, Edith C. Kieffer, Brandy Sinco, Gloria Palmisano, Michael S. Spencer, Gretchen A. Piatt

**Affiliations:** 1 University of Michigan School of Social Work, Ann Arbor, MI 48109, USA; ekieffer@umich.edu (E.C.K.); brsinco@umich.edu (B.S.); mspenc@uw.edu (M.S.S.); 2 Community Health and Social Services (CHASS), Detroit, MI 48209, USA; gpalmisano@chasscenter.org; 3 Department of Learning Health Sciences, University of Michigan Medical School, Ann Arbor, MI 48109, USA; piattg@umich.edu

**Keywords:** diabetes, community health worker intervention, Latino, older adults

## Abstract

Diabetes management for older Latino adults is complex, given a higher incidence of multiple coexisting medical conditions and psychosocial barriers to self-management. Community health workers (CHWs) may be effective in reducing these barriers. The REACH Detroit CHW randomized controlled intervention studies with Latino/as with diabetes found improvements in self-management behaviors and glucose control after participating in a CHW-led intervention. Using data from the REACH Detroit Partnership′s cohort 3, this study used descriptive statistics and multiple linear regression analyses to evaluate whether the six-month CHW intervention had a greater effect on older Latino/as (ages 55 and older) than younger participants between baseline and post-intervention follow-up at six months. There were significant intervention effects by age group that varied by outcome. Compared to a control group that received enhanced usual care, there were statistically significant intervention effects demonstrating greater self-efficacy scores 1.27 (0.23, 2.32); *p* < 0.05, and reductions in HbA1c 1.02 (−1.96, −0.07); *p* < 0.05, among older participants in the CHW intervention, and increases in diabetes support 0.74 (0.34, 1.13); *p* < 0.001; and understanding of diabetes management 0.39 (0.08, 0.70); *p* < 0.01 among younger participants.

## 1. Introduction

Type 2 diabetes (diabetes) has been, and continues to be, one of the major epidemics in the United States, representing a growing public health problem that affects racial and ethnic minority groups disproportionately [1]. The U.S. Centers for Disease Control and Prevention reports that ethnic minorities, among them Hispanics, are at a higher risk for diabetes than non-Hispanic whites, with a 2013–2015 age-adjusted prevalence of diabetes among Hispanics overall of 12.1%, (13.8% for Mexicans) compared to 7.4% of non-Hispanic whites (7.4%) [2], (https://www.cdc.gov/diabetes/pdfs/data/statistics/national-diabetes-statistics-report.pdf; pp. 2–3). The Hispanic Community Health Study/Study of Latinos, a prospective cohort study of Latinos from four major U.S. metropolitan areas from 2008–2011, found an overall diabetes prevalence in Hispanics of 16.9% in the U.S [3].

Today, individuals with diabetes are living much longer than in the past [4]. Diabetes may be different in older adults than in young adults both metabolically, and in therapy approach and needs [5,6,7]. Metabolically, older adults with type 2 diabetes tend to have a normal range of hepatic glucose production, impaired non-insulin mediated glucose uptake, and mediated blood flow impairment [8]. Older age groups also tend to have the highest rate of diabetes-related end stage renal disease [9].

Diabetes management of older adults is complex, given that this population has a higher incidence of multiple coexisting medical conditions that can impact management [10]. Clinically-related diabetes outcomes can be enhanced through routine primary care, specialty care services and effective self-management [11]. Most patients need on-going diabetes self-management support that aids individuals in implementing and sustaining behaviors needed to manage their diabetes [12]. However, most programs that focus on improving self-management and other factors—distress, support, depression, and self-efficacy—lack a tailored focus on the needs of older adults [5,13]. Often, older adults are under-represented in diabetes interventions because of misconceptions, and changes in cognitive and psychosocial characteristics that can affect diabetes self-care [5,14]. Thus, the best approach to providing diabetes self-management interventions for older adults has not been determined due to limited randomized controlled trial (RCT) data specific to older adults [14]. To our knowledge, only one published study has specifically compared the effectiveness of a behavioral intervention in improving glucose control among younger versus older adults (≥65 years) with poorly controlled blood sugar control [14]. The authors conducted a secondary analysis of age effects using data from a randomized controlled trial. They reported that while both younger adults and older adults can benefit from behavioral interventions, younger people showed greater improvement in self-care compared to older people at 3 and 6 months. The study population was 80.1% non-Hispanic white and conducted in a research intensive clinical setting.

Trained community health workers (CHWs) are particularly effective at providing diabetes-related support [15,16,17,18]. As trusted members of their respective communities, CHWs reach members of communities of color who face various barriers to diabetes self-management [19]. Examples include beliefs about the inevitability of diabetes due to family history; cultural and family food beliefs and practices; employment and child care responsibilities; age, weight and physical limitations affecting ability to be physically active; and health care providers who do not understand their language, culture or needs [20]. Diabetes self-management education and support for reducing diabetes risk factors, and Spanish-language materials and staff who understand their culture and needs were recommended by community residents. These recommendations led to the REACH Detroit Partnership’s CHW-focused intervention design [20]. Studies of these interventions with Latinos with diabetes consistently found overall improvements in self-management behaviors and blood sugar control [15,21], including a recent randomized controlled trial [22]. However, in the RCT [22], the intervention effects were not analyzed by age group. Given cognitive and psychosocial factors that may pose barriers to diabetes self-management among older adults [5,14], the research team hypothesized that the significant intervention effects of the parent study would be attenuated among older participants. We used data from the most recently completed REACH Detroit Partnership RCT to evaluate whether the CHW intervention was equally effective for older and younger Latino participants in improving diabetes self-management knowledge and behavior, diabetes self-efficacy, diabetes related distress, depressive symptoms and blood sugar control.

## 2. Materials and Methods 

### 2.1. Study Design and Population

The study analyzes data from the RCT conducted by the REACH Detroit Partnership that tested the effectiveness of a CHW intervention compared to an enhanced usual care (EUC) control group in Latinos with type 2 diabetes. The study origins, design, setting, population, recruitment and randomization procedures and intervention protocols have been described in depth elsewhere [22]. Briefly, the parent study was conducted between October 2009 and February 2013 with Latino adults with physician-diagnosed type 2 diabetes who were patients at CHASS Center, Inc., a federally qualified health center (FQHC) in southwest Detroit. Eligible participants were 21 years of age or older and self-identified as Latino/a. Those with physical and mental health limitations and illnesses, including substance use disorders preventing participation were excluded. Eligible patients participated in an orientation to the study. Those who consented to participate completed baseline data collection, followed by randomization using a computer-generated process with concealed allocation. The final study cohort included 222 participants randomized to the CHW intervention (*n* = 149) and the EUC (*n* = 73). The first follow-up data collection was conducted at 6 months, following completion of the intervention. It compares data that classified participants into 2 categories: older (55 years of age and older) or younger (those under age 55). The 55-year cut-point was determined after reviewing adequacy of power to detect significance effects for older participants, given their smaller sample size. This study uses data from the first follow-up. The University of Michigan Institutional Review Board approved the study.

### 2.2. Interventions

The CHW intervention used a culturally tailored diabetes self-management and healthy lifestyle curriculum called “Journey to Health”/El Camino a la Salud, which was developed using a community-based participatory research approach that has been described in-depth elsewhere [20,23]. The curriculum was not age- or gender-tailored. Additional details regarding the background and training of the CHWs, and the implementation of the intervention are also available [22]. Briefly, the CHW intervention was conducted by three Spanish-speaking Latina CHWs who were trained in CHW core competencies, diabetes, group process, empowerment theory and approaches, and motivational interviewing. The six-month long CHW intervention included 11 two-hour group meetings/classes conducted every two weeks, two 60-min home visits each month, accompanying the participant to a provider visit once (or more often, if requested) and phone calls as needed. The CHWs used empowerment approaches to discuss participant experiences and to help them to set and evaluate their self-management goals and action plans during the home visits and phone calls.

A graduate student research assistant conducted a two-hour class on how to interpret clinical and anthropometric results for EUC participants, and monthly phone calls to maintain updated contact information. Both the CHW intervention and EUC group participants also received usual health care at CHASS and referrals to community healthy lifestyle-related activities.

### 2.3. Study Measures

The primary outcome was Hemoglobin A1c (HbA1c) to assess blood sugar control. Hemoglobin A1c (HbA1c) is a measure of average blood sugar levels over a 3–6 month period, with higher scores indicating higher blood sugar levels. HbA1c was measured with a Bayer DCA 2000+ Analyzer [24]. Diabetes support was assessed by the Diabetes Support Scale (DSS), an instrument that assesses perceived social support related to meeting emotional needs, seeking advice, and obtaining information. The DSS ranges from one to six, where 1 indicates strongly disagreeing and 6 indicates strongly agreeing with being supported [25]. Understanding of diabetes self-management was computed from the mean of 16 questions from the Diabetes Care Profile, 1 = poor to 5 = excellent [26]. The Stanford Self-Efficacy for Diabetes scale was used to assess self-efficacy from a series of questions “How confident are you 1 to 10 …?” [27]. The Patient Activation Measure (PAM), a 0 to 100 scale that assesses patient knowledge, skill, and confidence for self-management, was used to evaluate patient activation [28]. We assessed diabetes-related distress using the Diabetes Distress Scale (DSS), a 17-item instrument that assesses emotional distress and functioning specific to living with diabetes. Scores under 2 indicate little or no distress; 2 to 3 corresponds to moderate distress; and above 3 indicates high distress [29]. Self-management behavior was evaluated via the Summary of Diabetes Self-Care Activities Scale, on a scale of 0 to 7 with a sequence of questions “On how many of the past 7 days did you …”. Sample items included testing blood sugar as recommended by one′s doctor, checking feet, taking prescribed diabetes pills or insulin, eating 5 or more servings of fruits and vegetables, and exercising for at least 30 minutes [30]. Depressive symptoms were assessed with the Patient Health Questionnaire-9 (PHQ-9). The PHQ-9 has the following interpretation: 1–4 minimal, 5–9 mild, 10–14 moderate, 15–19 moderately severe, 20–27 severe [31].

### 2.4. Statistical Analysis

All baseline characteristics were compared by a binary indicator for under age 55 years (younger) versus 55 years of age and older (older) within the EUC and CHW intervention groups. Our previous study includes the comparison of baseline characteristics between the EUC and CHW groups [22]. Categorical variables were compared between groups with the Pearson Χ^2^ test or with the Fisher exact test for rare outcomes. The Cochran-Armitage Trend test was used to compare ordinal categorical variables between groups [32,33]. The log-rank test was used to compare diabetes duration (years) between groups [34]. The *t*-test was used to compare means. All baseline comparison test results were checked for multiple comparison error by using Monte-Carlo simulation [35,36].

Outcomes were evaluated for intervention effects by using linear regression models. All regression models had the (6 month—baseline) change score as the outcome. The covariates were baseline value, treatment group (1 = CHW, 0 = EUC), a binary indicator for age 55 years and older, an interaction between treatment group and age 55 years and older indicator, gender (1 = male, 0 = female reference), high school education or more education (1 = at least a high school education, 0 = did not graduate), and employment (1 = employed full- or part-time, 0 = unemployed). These covariates were chosen because of differences between the EUC and CHW groups, overall, or by age within intervention group. The HbA1c model contained an additional indicator for diabetes medication intensification. Medication intensification was assessed by a binary indicator set to 1 if the dose or frequency of a particular diabetes medication increased from baseline to 6 months or if the number of diabetes medications increased. Otherwise, the diabetes medication intensification indicator was set to 0.

From the linear regression model, changes in HbA1c, self-management, and psychosocial outcomes from baseline to 6 months were estimated by post hoc contrasts by intervention group and by age group. For example, to estimate the average change in HbA1c for people aged 55 and older in the CHW intervention group, the treatment group indicator was set to 1 for CHW, the age group indicator was set to 1 for “≥55”, and the sum of regression coefficients was calculated for all covariates set their average values. More details are provided in Appendix A and Appendix B. Monte Carlo methods were used to adjust for multiple comparisons [37]. In addition to the adjusted comparisons from the regression models, we also computed unadjusted mean changes in HbA1c by age and intervention group, along with 95% confidence intervals.

For further exploration of independent variables associated with HbA1c change, penalized regression was conducted on HbA1c change with covariates of baseline value, demographic variables, CHW or EUC intervention, and interactions between independent variables and treatment group. Details are provided in Appendix C. All analyses were performed with SAS version 9.4 (Cary, NC, USA). 

## 3. Results

Table 1 compares the baseline characteristics of those younger than 55 years of age with those 55 years of age and older within the Enhanced Usual Care (EUC) group and within the Community Health Worker (CHW) intervention group. There were no significant sociodemographic differences by age group within the EUC group. Within the CHW group, younger participants were more likely to be female than older participants and to be employed. The percentage of participants reporting medication intensification was approximately three times higher in those 55 years of age and over compared to those under 55 years of age in the EUC group, but it was not significantly different in the CHW group. 

There were no significant baseline differences between age groups within either the EUC group or the CHW group in level of diabetes support, understanding of diabetes management, self-efficacy, patient activation or HbA1c. Within the EUC group, older participants had higher diabetes self-management behavior scores than younger participants. Within the CHW group, younger participants had higher diabetes distress scores than older participants, with little or no distress in older participants and moderate distress in younger participants. Similarly, in the CHW group, younger participants also had more depressive symptoms than older adults. 

Table 2 presents estimates of outcome changes between baseline and 6 months from linear regression models for each outcome, with covariates of indicators for intervention group and for age 55 years and older, their interaction, and adjusted for the characteristics that varied by age or intervention group at baseline. The HbA1c model contained an additional indicator for medication intensification. Within the EUC group, there were no significant changes from baseline for either younger or older participants for any outcome except understanding of diabetes management. Both age groups demonstrated a significant increase in this outcome. 

Within the CHW group, there were significant improvements for all outcomes, with some differences by age group. Diabetes support increased among, both participants under age 55 and among those ages 55 and older. There was a significant intervention effect in diabetes support for younger but not older CHW intervention participants, compared to the EUC group Similarly, understanding of diabetes management improved among both CHW intervention participants under age 55 and those ages 55 and older. There was a significant intervention effect in understanding of diabetes management for younger, but not older, participants in the CHW intervention group, compared to the EUC group.

Within the CHW group, self-efficacy improved for both younger and older participants. However, there was a significant intervention effect for older, but not younger, participants, with those ages 55 and older demonstrating significant increases in self-efficacy, compared to the EUC group.

Within the CHW group, HbA1c decreased by an average of −0.34 (−0.60, −0.08); *p* < 0.01 for those aged under 55, and by −0.83 (−1.25, −0.40); *p* < 0.001 for participants aged 55 and older. There was a significant intervention effect for the older participants in the CHW group, achieving a statistically and clinically significant decrease in HbA1c: −1.02 (−1.96, −0.07); *p* < 0.05, when compared to the EUC group. In contrast, the HbA1c intervention effect was not significant among younger participants. None of the results changed after the *p* values were recomputed with Monte Carlo simulation to account for multiple comparisons.

There were no significant intervention effects in patient activation scores, diabetes distress, self-management, or depressive symptoms in either age group.

**Figure A1:**
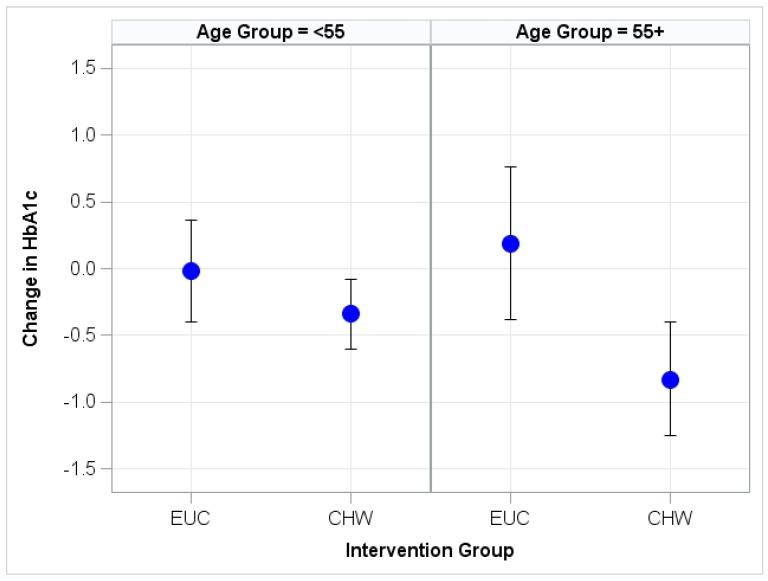
Adjusted Mean HbA1c Changes by Age and Intervention Group with 95% Confidence Intervals ^a,b^. ^a^ EUC = Enhanced Usual Care control group; CHW = Community Health Worker intervention group. ^b^ Adjusted intervention effect for participants under age 55: −0.32 (−0.93, 0.28), *p* = 0.503; intervention effect for participants ages 55+: −1.02 (−1.96, −0.07), *p* = 0.029. Regression outcome was the (6 month—baseline) difference score. Covariates were baseline value, treatment group, indicator for age ≥55, interaction between treatment group and age ≥55 indicator, gender, high school education, employment, and medication intensification.

In Appendix A, Figure A1 illustrates the mean changes in HbA1c by intervention group and by age category that are enumerated in Table 2. The unadjusted HbA1c comparisons led to the same conclusions as the adjusted regression model: significant HbA1c drops within the CHW group for both age groups and a significant intervention effect only for participants 55 years of age and older: −1.03 (−1.85, −0.22); *p* = 0.014. Appendix B documents the equations and SAS code used for the regression models. Appendix C displays the variable selection output using penalized regression, indicating that the most important predictors for HbA1c drop were baseline HbA1c, intervention group, and the interaction between intervention group and age group.

## 4. Discussion

This study analyzed whether a community health worker intervention tailored to the needs of Latinos with diabetes was equally effective among older (55 years of age and older) and younger adult participants. The intervention was not age-tailored. The results showed that the impact of age varied by outcome. Within the community health worker intervention group, older participants demonstrated significant changes for all outcomes except patient activation while younger participants demonstrated significant changes for all outcomes except self-management behavior. Within the enhanced usual care control group, the only outcome that changed significantly was understanding of diabetes management, which changed in both age groups. Compared to enhanced usual care, there were statistically significant intervention effects demonstrating greater self-efficacy scores and reductions in HbA1c among older participants in the community health worker intervention. Additionally, younger participants experienced improvements in diabetes support and understanding of diabetes management.

Achieving healthy levels of blood sugar control helps to prevent diabetes complications and associated functional disability specifically in older adults [38]. This study found that the significant intervention effect on HbA1c documented in our parent study [22], is most evident among participants aged 55 and older, with a clinically significant drop in HbA1c of −1.02 in that age group. To our knowledge, only one other study has evaluated the age effects of a diabetes intervention on HbA1c. In a study conducted primarily with non-Hispanic white (83.1%) and well educated (14.7 years of education) participants, Beverly et al. compared the effects of three highly structured diabetes education interventions (two group; one individual), comparing the age effects among adults aged 60–75 versus those of younger age [14]. They found that older and younger adults had equal improvements in HbA1c over a 12-month follow-up period [14]. Older adults benefited most from one of the two group interventions while younger adults in the individual intervention benefited more than older adults [14]. Beverly et al. found no significant age effects for other outcomes except reported self-care where younger participants showed greater improvement at six but not at 12 months [14]. Our study found no intervention effect for either age group in self-management behavior. 

A study of older adults with a diagnosis of diabetes from the California Health Interview Survey found that, among Latino participants, those with higher levels of diabetes self-efficacy demonstrated decreased levels of psychological distress [39]. In our study, the community health worker intervention had a significant intervention effect on self-efficacy among older Latino adults, but not on those under 55 years old. We did not test the age effect of improved self-efficacy and other outcomes. 

Providing ongoing diabetes education and self-management support aids in overcoming barriers and coping throughout treatment and other transformations [40]. Currently, there is a lack of literature that analyzes age effects on diabetes support and education, thus aging specific guidelines are challenging to present [41]. It might be expected that older adults need more support due to such factors as frailty, dementia and medication management challenges [42]. In the United States, current statistics demonstrate that isolation impacts up to 17% of older adults [43], which may pose a barrier to achieving adequate diabetes support. The results of this study showed that both older and younger Latinos showed improvements in reported diabetes support. However, there was a significant intervention effect only among younger participants. Results from this study also showed that although understanding of diabetes management improved in both age groups, significant intervention effects were only demonstrated among younger participants. 

There are several considerations and limitations to this study. These are similar to those described for the parent REACH Detroit study, including generalizability beyond urban Latinos using a federally qualified health center, exclusion of those unwilling or unable to participate, self-reported measures of behavioral and psychosocial outcomes, and limitations in sample size [22]. Our power to detect statistically significant changes in some outcomes may have been reduced due to age disaggregation, especially for the older age group. Nonetheless, the study was able to detect clinically and statistically significant improvements in several outcomes. Future research that includes a larger sample size of diverse Latinos from several locations is recommended to confirm these results. A strength of this study was its unique ability to evaluate the impact of a community health worker intervention among older and younger Latino participants. The community health worker intervention was not age-tailored. However, the intervention was largely conducted in a group setting, diverse with respect to age, goal setting and some individual contacts with community health workers, which may have allowed participants to address some age-related issues. Future research may help indicate if tailoring interventions to older participants in a group intervention would foster or impair achieving successful outcomes.

## 5. Conclusions

In the next 20 years the number of older adults with diabetes is projected to double [10]. Additionally, the National Health Council on Aging reported that there were 3.6 million older adult Hispanics in 2014. They made up around 8% of the aging population in America, with numbers projected to increase to 21.5 million by 2060, rising to 22% of the older adult population in the United States [44]. Despite these demographic trends, there is still limited literature regarding Latinos and age effects on diabetes-related outcomes, including the effects of interventions. 

Older Latino participants with diabetes in the community health worker-led arm of our randomized controlled trial achieved clinically significant improvements in self-efficacy and blood sugar control. These results suggest that while a general community health worker-led diabetes intervention may help participants achieve important outcomes, considering the issues and problems of older adults may be important considerations for both future research and implementation of similar interventions in clinical and community settings.

## Figures and Tables

**Table 1:** Baseline Characteristics (REACH-Detroit Cohort 3, *N* = 222).

Intervention Group	Enhanced Usual Care (*n* = 73)			Community Health Worker (*n* = 149)		
Age Group	<55 Years (*N* = 53)	≥55 Years (*N* = 20)	*p*-Value	<55 Years (*N* = 105)	≥55 Years (*N* = 44)	*p*-Value
**Demographic Characteristics**						
Female, *n* (%)	34 (64.2%)	15 (75.0%)	0.379 ^a^	66 (62.9%)	20 (45.5%)	0.049 ^a^
Spanish speaking, *n* (%)	44 (83.0%)	17 (85.0%)	>0.999 ^b^	87 (82.9%)	41 (93.2%)	0.124 ^b^
≥High School Education, *n* (%)	26 (49.1%)	6 (30.0%)	0.143 ^a^	27 (25.7%)	9 (20.5%)	0.494 ^a^
Employed, *n* (%)	24 (45.3%)	8 (40.0%)	0.685 ^a^	50 (47.6%)	13 (29.5%)	0.042 ^a^
Place of Birth			0.751 ^b^			0.055 ^b^
Mexico	33 (63.5%)	12 (60.0%)		74 (71.2%)	33 (75.0%)	
Other Latin American Country	10 (19.2%)	3 (15.0%)		18 (17.3%)	2 (4.5%)	
US	9 (17.3%)	5 (25.0%)		12 (11.5%)	9 (20.5%)	
US residence for ≥20 years, *n* (%)	20 (38.5%)	11 (55.0%)	0.204 ^a^	51 (48.6%)	20 (45.5%)	0.728 ^a^
Married or partnered, *n* (%)	40 (75.5%)	12 (60.0%)	0.193 ^a^	77 (73.3%)	27 (61.4%)	0.147 ^a^
**Health Related Characteristics**						
Diabetes duration (years), mean (sd)	4.42 (4.99)	7.56 (5.54)	0.055 ^c^	5.60 (5.54)	8.00 (6.48)	0.037 ^b^
Antihyperglycemic medication, *n* (%)			>0.999 ^d^			0.585 ^d^
No medications	3 (5.7%)	0 (0.0%)		6 (5.7%)	1 (2.3%)	
Only oral diabetes medication	34 (64.2%)	15 (75.0%)		73 (69.5%)	36 (81.8%)	
Insulin, with or without medication	16 (30.2%)	5 (25.0%)		26 (24.8%)	7 (15.9%)	
Medication Intensification ^e^, *n* (%)	8 (15.1%)	9 (45.0%)	0.012 ^b^	32 (30.5%)	12 (27.3%)	0.844 ^b^
**Outcome Values at Baseline**						
Diabetes Support, mean (sd) ^g^	3.97 (1.16)	4.06 (1.01)	0.749 ^f^	4.25 (1.04)	4.02 (1.13)	0.233 ^f^
Understanding of Diabetes Management ^h^, mean (sd)	2.79 (0.90)	2.75 (0.85)	0.842 ^f^	2.80 (0.84)	2.79 (0.72)	0.952 ^f^
Self-Efficacy ^i^, mean (sd)	7.11 (1.72)	7.61 (1.48)	0.249 ^f^	6.82 (1.71)	7.38 (1.52)	0.058 ^f^
Patient Activation Measure ^j^ (PAM), mean (sd)	56.03 (10.38)	56.51 (7.31)	0.829 ^f^	56.90 (8.36)	55.14 (4.19)	0.090 ^f^
Diabetes Distress ^k^, mean (sd)	2.08 (0.93)	1.89 (1.01)	0.462 ^f^	2.26 (1.11)	1.69 (0.65)	<0.001 ^f^
Self-Management Behavior ^l^, mean (sd)	3.23 (1.18)	4.02 (0.94)	0.009 ^f^	3.32 (1.29)	3.64 (1.06)	0.144 ^f^
Depressive Symptoms ^m^, mean (sd)	5.04 (3.78)	4.30 (5.45)	0.583 ^f^	6.88 (5.98)	4.16 (3.70)	0.001 ^f^
HbA1c, mean (sd)	7.83 (1.92)	7.40 (1.37)	0.365 ^f^	8.01 (2.07)	7.60 (1.47)	0.171 ^f^

^a^ Pearson Chi-Square Test, ^b^ Fisher Exact Test, ^c^ Log-Rank Test, ^d^ Cochran-Armitage Trend test. ^e^ Medication Intensification = Higher dose or frequency of same diabetes medications, or addition of new diabetes medication from baseline to 6-months. ^f^ T-Test. ^g^ Diabetes Support Scale (DSS). 1 = Strongly Disagree to 6 = Strongly Agree. ^h^ Diabetes Care Profile; 1 = Poor to 5 = Excellent. ^i^ Stanford Self-Efficacy for Diabetes “How confident are you 1 to 10 …?”. ^j^ Patient Activation Measure; 0–100 scale. ^k^ Diabetes Distress Scale (DDS). <2: little or no distress; 2 to <3: moderate distress; ≥3: high distress. ^l^ Summary of Diabetes Self-Care Activities”, “On how many of the past 7 days did you …?” ^m^ Interpretation of depression level from the Patient Health Questionnaire (PHQ-9): 1–4 minimal, 5–9 mild, 10–14 moderate, 15–19 moderately severe, 20–27 severe.

**Table 2:** Baseline to 6-Month Changes in Outcomes and Intervention Effects from Linear Regression Models ^a^, Mean (95% Confidence Intervals).

Outcome	Age Group	EUC ^a,b^ Change	CHW ^a,b^ Change	Intervention Effect ^c^
Diabetes Support ^d^	<55	0.10 (−0.15, 0.35)	0.83 (0.67, 1.00) ***	0.74 (0.34, 1.13) ***
	≥55	0.32 (−0.05, 0.69)	0.87 (0.60,1.15) ***	0.56 (−0.05, 1.17)
Understanding of Diabetes Management ^e^	<55	0.19 (0.0002, 0.38) *	0.58 (0.45, 0.71) ***	0.39 (0.08, 0.70) **
	≥55	0.36 (0.08, 0.65) *	0.38 (0.17, 0.59) ***	0.02 (−0.46, 0.49)
Self-Efficacy ^f^	<55	0.34 (−0.08, 0.76)	0.62 (0.33, 0.91) ***	0.28 (−0.40, 0.96)
	≥55	−0.33 (−0.96, 0.31)	0.95 (0.48, 1.41) ***	1.27 (0.23, 2.32) *
Patient Activation Measure ^g^ (PAM)	<55	2.42 (−0.13, 4.98)	4.45 (2.72, 6.19) ***	2.03 (−2.06, 6.11)
	≥55	1.11 (−2.71, 4.93)	0.62 (−2.20, 3.44)	−0.49 (−6.79, 5.81)
Diabetes Distress ^h^	<55	−0.06 (−0.30, 0.17)	−0.36 (-0.52, −0.20) ***	−0.29 (−0.67, 0.08)
	≥55	−0.16 (−0.51, 0.19)	−0.31 (-0.57, −0.05) *	−0.15 (−0.72, 0.42)
Self-Management Behavior ^i^	<55	−0.13 (−0.46, 0.21)	0.19 (−0.04, 0.42)	0.32 (−0.22, 0.86)
	≥55	−0.31 (−0.82, 0.19)	0.40 (0.03, 0.77) *	0.72 (−0.11, 1.54)
Depressive Symptoms ^j^	<55	−0.37 (−1.55, 0.80)	−0.87 (−1.67, −0.06) *	−0.49 (−2.40, 1.41)
	≥55	−1.62 (−3.37, 0.12)	−1.47 (−2.77, −0.18) *	0.15 (-2.73, 3.03)
HbA1c	<55	−0.02 (−0.40, 0.36)	−0.34 (−0.60, −0.08) **	−0.32 (−0.93, 0.28)
	≥55	0.19 (−0.38, 0.76)	−0.83 (−1.25, −0.40) ***	−1.02 (−1.96, −0.07) *

^a^ Regression outcome was the (6 month—baseline) difference score. Covariates were baseline value, treatment group, indicator for age ≥ 55, interaction between treatment group and age ≥ 55 indicator, gender, high school education, and employment. HbA1c model contained additional indicator for diabetes medication intensification. ^b^ EUC = Enhanced Usual Care control group; CHW = Community Health Worker intervention group. In the EUC column, the p values refer to the (6 months—baseline) difference score within the EUC group. In the CHW column, the p values refer to the (6 month—baseline) change within the CHW group. ^c^ Intervention effect = (6 months—baseline) difference score within CHW minus EUC; Intervention effect = CHW − EUC. ^d^ Diabetes Support Scale (DSS). 1 = Strongly Disagree to 6 = Strongly Agree. ^e^ Diabetes Care Profile; 1 = Poor to 5 = Excellent. ^f^ Stanford Self-Efficacy for Diabetes “How confident are you 1 to 10 …?”. ^g^ Patient Activation Measure; 0–100 scale. ^h^ Diabetes Distress Scale (DDS). <2: little or no distress; 2 to <3: moderate distress; ≥3: high distress. ^i^ Summary of Diabetes Self-Care Activities”, “On how many of the past 7 days did you …?” ^j^ Interpretation of depression level from the Patient Health Questionnaire (PHQ-9): 1–4 minimal, 5–9 mild, 10–14 moderate, 15–19 moderately severe, 20–27 severe. * *p* < 0.05; ** *p* < 0.01; *** *p* < 0.001.

**Figure A2:**
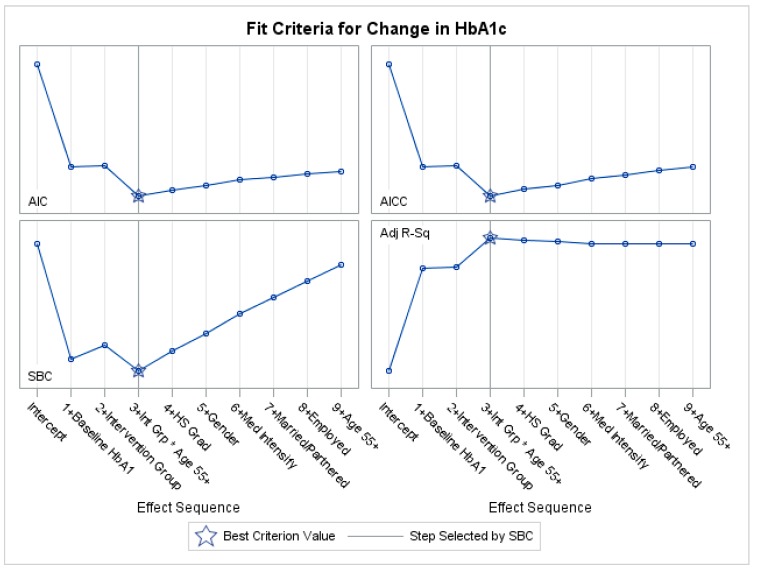
Selection of Predictors for Baseline to 6-Month Change in HbA1c by Using the LASSO ^a^ Method with (Akaike Information Criteria) AIC, (Bayesian Information Criteria) SBC, and Adjusted R-Squared Criteria. ^a^ LASSO = Least Absolute Shrinkage and Selection Operator.

## Appendix B. Linear Regression Model and SAS Code

- Let BL_HbA1c = Hemoglobin HbA1c at baseline.
- Let M6_HbA1c = Hemoglobin HbA1c at 6 months.
- Let M6BL_HbA1c = M6_HbA1c–BL_HbA1c.
- Let GenderInd = 1 if male, 0 if female (reference).
- Let HSGrad =1 if high school graduate, 0 if less than high school education (reference).
- Let EmpBin =1 if employed full or part-time; 0 if unemployed (reference).
- Let M6_MoreDiabetesMeds = 1 if medication intensification, 0 if not (reference).
- Let RandomizationBinN = −1 if intervention group, 0 if control group (reference); (RandomizationBinN = −1) is 1 if RandomizationBinN = −1 & 0 if RandomizationBinN = 0.
- Let AgeGE55 = −1 if age ≥55, 0 if age <55 (reference); (AgeGE55N = −1) is 1 if AgeGE55N = −1 & 0 if AgeGE55N = 0.

Note: −1 and 0 coding were used because SAS sets the reference category in a class statement to the highest value. Using −1 instead of 1 will set the reference category to 0. SAS will create binary indicators for each category of variables in the class statement.

- β_0_–β_7_ are regression coefficients.
- ε = random error.

**Regression Equation for HbA1c Change:**

M6BL_HbA1c = β_0_ + β_1_ × BL_HbA1c + β_2_ × GenderInd + β_3_ × HSGrad + β_4_ × EmpBin + β_5_ × M6_MoreDiabMeds + β_6_ × (RandomizationBinN = −1) + β_7_ × (AgeGE55N = −1) + β_8_ × (RandomizationBinN = −1) × (AgeGE55N = −1) + ε.

SAS Code for Linear Regression and Post-Hoc Estimates

/* Estimate statements were used to compute intervention effects by computing the difference between the regression coefficients for the intervention and control groups. */

/* LSMeans stands for “least squares means”. This statement was used to compute within-group comparisons by age and intervention group. In the LSMeans statement, all covariates, other than the class variables, are set to their average values in the data set. */

proc GLM data = DiabetesDetroit;

Class RandomizationBinN AgeGE55N;

model M6BL_HbA1c = BL_HbA1c GenderInd HSGrad EmpBin M6_MoreDiabMeds RandomizationBinN AgeGE55N RandomizationBinN*AgeGE55N/solution;

Estimate ‘Int Eff AgeGE55-Age < 55’ RandomizationBinN*AgeGE55N 1 −1 −1 1

Estimate ‘Int:Ctl AgeGE55’ RandomizationBinN 1 −1 RandomizationBinN*AgeGE55N 1 0 −1 0;

Estimate ‘Int:Ctl Age < 55’ RandomizationBinN 1 −1 RandomizationBinN*AgeGE55N 0 1 0 −1;

**/* Proc GLM can adjust for multiple comparisons, using Monte Carlo simulation, in the LSMeans statement. */**

LSMeans RandomizationBinN*AgeGE55N/StdErr CL PDIFF Adjust = Simulate (NSAMP = 10000 SEED = 5072018);

Store RegM6BL_HbA1c; run; quit;

**/* Use Proc PLM to adjust the quantities in the Estimate statement for multiple comparisons, using Monte Carlo simulation. */**

PROC PLM Restore = RegM6BL_HbA1c;

Estimate ‘Int Eff AgeGE55-Age < 55’ RandomizationBinN*AgeGE55N 1 −1 −1 1 / adjust = simulate (NSAMP = 10000 SEED = 5072018);

Estimate ‘Int:Ctl AgeGE55’ RandomizationBinN 1 −1 RandomizationBinN*AgeGE55N 1 0 −1 0/ adjust = simulate(NSAMP = 10000 SEED = 5072018);

Estimate ‘Int:Ctl Age < 55’ RandomizationBinN 1 −1 RandomizationBinN*AgeGE55N 0 1 0 −1/ adjust = simulate(NSAMP = 10000 SEED = 5072018);

ODS Output Estimates = PLM_M6BL_HbA1c;

Run;

Proc Print Data = PLM_M6BL_HbA1c; Run;

## Appendix C. Variable Selection with Penalized Regression

For further exploration of independent variables associated with HbA1c change, penalized regression was conducted on HbA1c change with covariates of baseline value, demographic variables, CHW or EUC intervention, and interactions between independent variables and treatment group. Penalized regression includes penalties for unnecessary variables and is a state of the art alternative to stepwise regression. The model with best subset of variables for prediction will have the lowest information criteria and the maximum adjusted R-squared. The penalized regression methods used for variable selection were LASSO and Elastic Net [45,46,47]. LASSO stands for Least Absolute Shrinkage and Selection Operator. Both LASSO and Elastic Net are algorithms for identifying important predictor variables, while shrinking the coefficients for unimportant variables to zero [45,46,47].

Figure A2 displays the variable selection output using penalized regression with the LASSO technique. The LASSO analysis confirms the same result as Elastic Net, that the most important predictors for HbA1c drop were baseline HbA1c, intervention group, and the intervention between intervention group and age group. Regardless of which information criteria was used to select the best combination of predictors, the best combination of variables was consistently identified as these three variables.
